# Influence of Nanoscale Inhomogeneity Incorporating Interface Effect on Crack Nucleation at Intersection of Twin and Grain Boundary in Nanocomposite

**DOI:** 10.3390/ma14216718

**Published:** 2021-11-08

**Authors:** Yongshu Tao, Liang Li, Guanghong Miao, Jilei Dong

**Affiliations:** School of Mechanics and Photoelectric Physics, Anhui University of Science and Technology, Huainan 232001, China; ll4714052@163.com (L.L.); miaogh@mail.ustc.edu.cn (G.M.); dongjilei0801@163.com (J.D.)

**Keywords:** nanocomposite, crack, twin, nanoinhomogeneity, interface effect

## Abstract

Nanocracks can generate at the intersection of the deformation twin and grain boundary (GB). A mathematical model is built to study the nanoinhomogeneity effect on nanocrack nucleation and propagation in the nanocrystalline matrix. The boundary condition at the interface between the nanoinhomogeneity and the matrix is modified by incorporating the interface effect. The influence of the nanoinhomogeneity shear modulus, the nanoinhomogeneity radius, the nanoinhomogeneity position, the interface effect, and the external stress on the nanocrack nucleation and propagation is investigated in detail. The results indicate that the stiff nanoinhomogeneity suppresses nanocrack nucleation and propagation and thereby improves the tensile ductility of nanocomposites without loss of their predominantly high strength. Both the positive interface residual tension and interface elastic constants suppress nanocrack nucleation and propagation, while the negative interface residual tension and interface elastic constants promote nanocrack nucleation and propagation. Furthermore, the effect of interface residual tension is rather significant. The interface elastic constants have a weak effect on nanocrack nucleation and propagation.

## 1. Introduction

Nanocrystalline materials showing superior strength and hardness have recently attracted a considerable amount of attention [1,2,3,4,5,6]. At the same time, in most cases, nanocrystalline materials exhibit disappointingly low tensile ductility and low fracture toughness, which substantially limit their practical application [7,8,9,10]. Usually, the fracture of nanocrystalline materials often begins with nanocrack generation at grain boundaries and their triple junctions [11,12,13,14]. Structural defects and stress concentrations located at grain boundaries are the most probable sites of nanocrack nucleation [15]. For instance, the disclinations created by GB sliding serve as powerful stress sources that can induce the generation of nanocracks, and then the nanocrystalline materials tend to exhibit brittle behavior [13,14]. Many previous works have studied the crack generation from disclinations [16,17,18].

In addition, fracture and twin deformation often occur cooperatively in nanocrystalline materials. For example, the pre-existent crack can stimulate the generation of a deformation twin in nanocrystalline materials [19]. Furthermore, molecular dynamics simulations [20] have shown that the deformation twin also promotes crack generation because of the high local stress produced from the intersection between the twin and grain boundary in nanocrystalline Mo. The molecular dynamics simulations [20] are in good agreement with the experiments [21,22] documenting cracks can nucleate at twin–grain boundary intersections in coarse-grained γ -TiAl. Based on the above description, Ovid’ko and Sheinerman [23,24] built a theoretical model to study crack nucleation at the intersection of the twin and grain boundary in thin-film and bulk nanocrystalline materials. They found that the nanocrack is produced at twin thicknesses of a few nanometers, and the equilibrium nanocrack lengths increase with increasing twin thickness in bulk nanomaterials. Besides, the influence of the film surface on nanocrack nucleation is significant in thin-film nanomaterials, when the distance between the film and the twin surface is less than several twin thicknesses. Recently, Luo [25] investigated microcrack generation at the intersection between the twin and GB. The deformation twin is represented as a wedge disclination quadrupole. They found that when the aspect ratio of the twin is large, the effect of the distant wedge disclination dipole can be neglected, so the disclination dipole model is sufficiently precise to investigate the crack generation.

In contrast with single-phase nanocrystalline materials, nanocomposites have high strength and crack resistance characteristics, which makes them highly attractive for a wide range of engineering applications [26,27,28,29,30,31,32,33]. The mechanical properties of Ni/SiC nanocomposites produced by pulse electrodeposition have been studied by Zimmerman et al. [30]. It was interesting to note that not only the tensile strength but also the ductility of the nanocomposites with 400 nm SiC particles were higher than those of pure nanocrystalline Ni materials. However, at higher SiC concentrations, the ductility and strength of the nanocomposites decreased significantly due to particle clustering. Lari Baghal et al. [33] have recently reported the experimental results of second-phase SiC nano-particles incorporated in electrodeposited nanocrystalline Ni-Co. They found that uniformly distributed SiC nanoparticles can significantly improve the ultimate tensile strength and elongation to failure of the nanocomposites.

For nanoscale inhomogeneity, with a large volume ratio of the interface area to the inhomogeneity, the interface effect on the property of the material cannot be neglected. Gurtin and his co-workers [34,35] first studied the interface stress of elastic isotropic solids with continuum mechanics and proposed the interface stress model. The interface stress model has been widely utilized to investigate the various mechanical problems in nanostructured materials [36,37,38,39,40,41,42,43,44,45]. Fang and Liu [38,39] studied the interaction of nanoinhomogeneity with screw dislocation and edge dislocation, respectively. Mogilevskayaet et al. [40] studied the interaction between a circular inclusion and a straight crack with interface elasticity and tension. They discussed the interface effect on the stress intensity factor at the crack tip by using a complex boundary integral equation approach. The numerical results showed that the interface tension may significantly change the stress intensity factor, while the effect of surface elasticity is rather insignificant. Luo et al. [41] investigated the stress field and crack nucleation behavior in a disclinated nanowire with the Gurtin–Murdoch model. They found that the stress fields of the wedge disclination and the edge dislocation are significantly affected by the surface effect. Zhu and Ju [42] investigated the effective elastic moduli of composite materials containing randomly distributed nanoparticles by incorporating the interface energy effect into a classical micromechanics framework. While there has been much recent work on nanocomposites, there is little systematic work on the influence of second-phase nanoparticles in the nanocrystalline matrix. The systematic studies of both stiff and soft second-phase nanoparticles with varying sizes and distributions in the nanocrystalline matrix are likely to provide additional optimization of strength and ductility in nanocomposite materials.

The main aim of this paper is to theoretically describe the nanoinhomogeneity effect on nanocrack nucleation and propagation at the deformation twin stopped by GBs in nanocomposite materials. The boundary condition at the interface between the nanoinhomogeneity and the matrix is modified by incorporating the interface effect. The influence of the nanoinhomogeneity shear modulus, the nanoinhomogeneity radius, the nanoinhomogeneity position, the interface effect, and the external stress on the nanocrack nucleation and propagation is discussed.

## 2. Model and Problem Formulation

**Figure 1:**
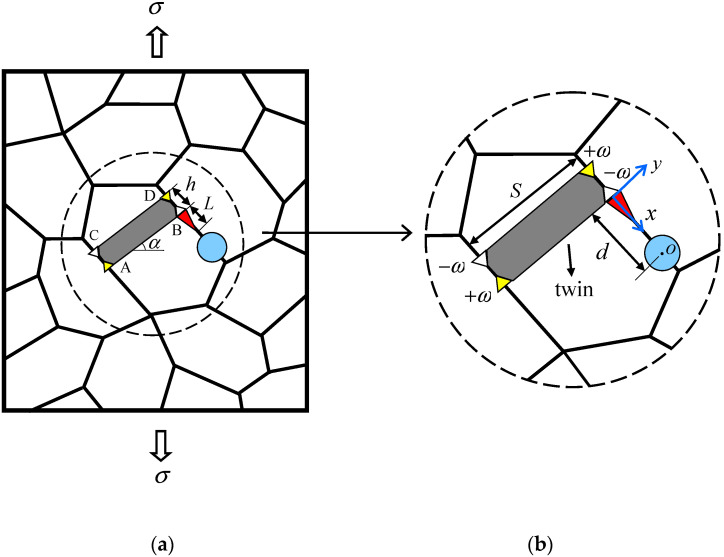
The nanocrack and nanoinhomogeneity at the nanoscale twin in a nanocomposite material. (**a**) Full view. (**b**) Magnified inset highlights the nanoscale twin, the nanocrack, and the nanoinhomogeneity.

As shown in Figure 1, a nanocrystalline matrix with the elastic properties $\mu_{2}$ and $\kappa_{2}$ contains a circular nanoinhomogeneity of the radius R with the elastic properties $\mu_{1}$ and $\kappa_{1}$, where $\mu_{j}$ (j=1,2) is the shear modulus and $\kappa_{j}=3-4\upsilon_{j}$ for the plane strain state ($\upsilon_{j}$ is Poisson’s ratio). The center of the circular nanoinhomogeneity o is assumed for convenience to locate the grain boundary. The nanocrystalline matrix is under a remote tension load σ.

According to Ovid’ko and Sheinerman [23], accompanying plastic deformation of the nanocrystalline matrix, the twin ABCD is produced in a nanoscale grain. To simplify the analysis, we suppose that the deformation twin ABCD is perpendicular to the grain boundary that stops its growth. Therefore, following Romanov and Vladimirov [46] and Ovid’ko and Sheinerman [23], the twin ABCD can be represented as a wedge disclination quadrupole. Furthermore, in the fcc crystals, the magnitude of the wedge disclination strength ω is $2\arctan(\sqrt{2}/4)$. The thickness and length of the twin are given as h and s, while the distance between point B and the circular nanoinhomogeneity center o is denoted as d.

Due to the high internal stress fields induced by disclinations, they often serve as the sites for crack nucleation. As shown in Figure 1, a nanocrack with length L is generated from one of the negative wedge disclinations and lies alongside the GB. Under the assumption that the twin length s is large compared with both the nanocrack length L and the twin thickness h, the influences of disclinations located at points A and C on the nanocrack nucleation and propagation can be ignored. That is to say, we studied the nucleation and propagation of the nanocrack in the stress field induced by the external load, the nanoinhomogeneity, and the disclination dipole.

According to Gurtin and Murdoch [34], the elastic field of the bulk solid can use classic elasticity to characterize the differential equation, while the interface between the nanoinhomogeneity and the matrix has its own elastic constants and is described by a supplementary constitutive relation. Following the work of Sharma et al. (2003) and assuming that the interface adheres to the nanoinhomogeneity without slipping, the boundary conditions on the nanoinhomogeneity-–matrix interface can be obtained as

(1) $$u_{x1}^{+}(t)-u_{x2}^{-}(t)=0,u_{y1}^{+}(t)-u_{y2}^{-}(t)=0,\left|t\right|=R$$

(2) $$\sigma_{rr1}^{+}(t)-\sigma_{rr2}^{-}(t)=-\frac{\sigma_{\theta \theta }^{0}(t)}{R},\sigma_{r\theta 1}^{+}(t)-\sigma_{r\theta 2}^{-}(t)=\frac{1}{R}\frac{\partial \sigma_{\theta \theta }^{0}(t)}{\partial \theta },\left|t\right|=R$$

where $\mu_{x}$ and $\mu_{y}$ are displacements in the x and y directions; the subscript ‘1’ indicates the nanoinhomogeneity and the subscript ‘2’ indicates the matrix; the superscripts + and − indicate boundary values of the physical quantity as z approaches the interface from the nanoinhomogeneity and the matrix, respectively; the superscript “0” indicates the interface area; and $\sigma_{rr}$ and $\sigma_{r\theta }$ are stress components in the polar coordinate. What is more, in light of Povstenko [47], the constitutive equation for the interface area is obtained as follows:

(3) $$\sigma_{\theta \theta }^{0}(t)=\tau^{0}+(2\mu^{0}+\lambda^{0}-\tau^{0})\varepsilon_{\theta \theta }^{0}(t)$$

where $\varepsilon_{\theta \theta }^{0}$ and $\sigma_{\theta \theta }^{0}$ indicate interface strain and stress, $\tau^{0}$ is the interface residual tension, and $\mu^{0}$ and $\lambda^{0}$ are interface elastic constants. Here we study a coherent interface, so the interface strain $\varepsilon_{\theta \theta }^{0}$ is equal to the associated tangential strain in the abutting bulk materials.

Taking into consideration the added constitutive equation for the interface area in Equation (3) and the following constitutive equation for the nanoinhomogeneity:

(4) $$\varepsilon_{\theta \theta 1}=\frac{\lambda_{1}+2\mu_{1}}{4\mu_{1}(\lambda_{1}+\mu_{1})}\sigma_{\theta \theta 1}-\frac{\lambda_{1}}{4\mu_{1}(\lambda_{1}+\mu_{1})}\sigma_{rr1}$$

the stress boundary conditions in Equation (2) on the interface are recast as follows

(5) $$\sigma_{rr1}^{+}(t)-\sigma_{rr2}^{-}(t)=-\frac{2\mu^{0}+\lambda^{0}-\tau^{0}}{4R\mu_{1}(\lambda_{1}+\mu_{1})}\left[\left(\lambda_{1}+2\mu_{1})\sigma_{\theta \theta 1}(t)-\lambda_{1}\sigma_{rr1}(t\right)\right]-\frac{\tau^{0}}{R}$$

(6) $$\sigma_{r\theta 1}^{+}(t)-\sigma_{r\theta 2}^{-}(t)=\frac{2\mu^{0}+\lambda^{0}-\tau^{0}}{4R\mu_{1}(\lambda_{1}+\mu_{1})}\left[(\lambda_{1}+2\mu_{1})\frac{\partial \sigma_{\theta \theta 1}(t)}{\partial \theta }-\lambda_{1}\frac{\partial \sigma_{rr1}(t)}{\partial \theta }\right]$$

where $\lambda_{1}$ is the Lame constant of the nanoinhomogeneity.

## 3. Conditions for Nanocrack Nucleation and Propagation

In order to estimate the conditions for nanocrack nucleation and propagation, we use the energetic criterion [48]

(7) $$F>2\gamma -\gamma_{b}$$

where F is the energy release rate, γ is the specific surface energy, and $\gamma_{b}$ is the specific (per unit area) GB energy.

According to Indenbom [48], the energy release rate of nanocrack propagation is obtained as

(8) $$F=\frac{\pi (1-\nu )L}{4\mu_{2}}({\bar{\sigma }}_{yy}^{2}+{\bar{\sigma }}_{xy}^{2})$$

where ${\bar{\sigma }}_{yy}$ and ${\bar{\sigma }}_{xy}$ are the mean weighted values of the stress tensor calculated as [48]

(9) $${\bar{\sigma }}_{iy}=\frac{2}{\pi L}\int_{0}^{L}\sigma_{iy}(x,y=0)\sqrt{\frac{x}{L-x}}dx,i=x,y.$$

$\sigma_{yy}$ and $\sigma_{xx}$ are the stress components of the total stress induced by the external load, the nanoinhomogeneity, and the disclination dipole BD.

According to the work of Fang and Liu [38] and Liu et al. [49], by a sufficient number of calculations, we can obtain the stress components $\sigma_{yy}$ and $\sigma_{xx}$ fitting into Formula (9) as

$$\sigma_{yy}(x,y=0)=[\frac{R^{2}}{{\left(d-x\right)}^{2}}+2]\{\frac{\sigma }{4}+\frac{D\omega }{2}\ln\frac{x+h}{x}+\frac{\mu_{2}}{\kappa_{2}\mu_{1}}\sum_{k=1}^{+\infty }B_{-k}{\left(d-x\right)}^{-k}\;-\frac{D\omega }{2\kappa_{2}}[\ln\frac{d-x-R^{2}/d}{d-x-R^{2}/(d+h)}-\frac{h}{d-x}+\frac{d+h-R^{2}/(d+h)}{d-x-R^{2}/(d+h)}-\frac{d-R^{2}/d}{d-x-R^{2}/d}]\}$$

$$+(d-x-\frac{R^{2}}{d-x})\{\frac{D\omega }{2}(\frac{1}{x}-\frac{1}{x+h})-\frac{\mu_{2}}{\kappa_{2}\mu_{1}}\sum_{k=0}^{+\infty }kB_{-k}{\left(d-x\right)}^{-k-1}$$

$$-\frac{D\omega }{2\kappa_{2}}[\frac{1}{d-x-R^{2}/d}-\frac{1}{d-x-R^{2}/(d+h)}+\frac{h}{{\left(d-x\right)}^{2}}-\frac{d+h-R^{2}/(d+h)}{{\left(d-x-R^{2}/(d+h)\right)}^{2}}+\frac{d-R^{2}/d}{{\left(d-x-R^{2}/d\right)}^{2}}]\}\;+\frac{R^{2}}{{\left(d-x\right)}^{2}}\{\frac{\kappa_{1}\mu_{2}}{\mu_{1}}\sum_{k=0}^{+\infty }A_{k}{\left(\frac{R^{2}}{d-x}\right)}^{k}-\frac{\mu_{2}}{\mu_{1}}A_{0}-\kappa_{2}[\frac{\sigma }{4}+\frac{D\omega }{2}\ln\frac{R^{2}/(d-x)-(d+h)}{R^{2}/(d-x)-d})]$$

(10) $$+\frac{D\omega }{2}[\ln\frac{R^{2}/(d-x)-R^{2}/d}{R^{2}/(d-x)-R^{2}/(d+h)}-\frac{h(d-x)}{R^{2}}+\frac{d+h-R^{2}/(d+h)}{R^{2}/(d-x)-R^{2}/(d+h)}-\frac{d-R^{2}/d}{R^{2}/(d-x)-R^{2}/d}]\}$$

$$\sigma_{xy}(x,y=0)=\frac{R^{2}}{{\left(d-x\right)}^{2}}(\frac{\mu_{2}}{\kappa_{2}\mu_{1}}\frac{1+\kappa_{2}}{\kappa_{2}c_{7}}\frac{R^{2}}{{\left(d-x\right)}^{2}}\frac{\sigma }{2}-\frac{R^{2}}{{\left(d-x\right)}^{2}}\frac{\sigma }{2\kappa_{2}}-\frac{\kappa_{1}\mu_{2}}{\mu_{1}}\frac{c_{9}}{c_{8}}\frac{R^{2}}{{\left(d-x\right)}^{2}}\frac{\sigma }{2}-\frac{{\left(d-x\right)}^{2}}{R^{2}}\frac{\sigma }{2})$$

(11) $$-(d-x-\frac{R^{2}}{d-x})(\frac{\mu_{2}}{\kappa_{2}\mu_{1}}\frac{1+\kappa_{2}}{\kappa_{2}c_{7}}\frac{R^{2}}{{\left(d-x\right)}^{3}}\sigma -\frac{R^{2}}{{\left(d-x\right)}^{3}}\frac{\sigma }{\kappa_{2}})$$

where

$$A_{0}=\frac{(1+\kappa_{2})\mu_{1}}{\kappa_{1}\mu_{2}-\mu_{2}+2\mu_{1}(1+b)}\frac{\sigma }{4}+\frac{D\omega }{2}\frac{c_{1}}{1-c_{2}}\ln\frac{d+h}{d}-\frac{\mu_{1}}{\kappa_{1}\mu_{2}-\mu_{2}+2\mu_{1}(1+b)}\frac{\tau^{0}}{R},\;A_{k}=-\frac{D\omega }{2}\frac{c_{4}}{c_{3}}R^{-2k}\{\frac{1}{k}[{\left(\frac{R^{2}}{d+h}\right)}^{k}-{\left(\frac{R^{2}}{d}\right)}^{k}]-h\delta_{1k}+(d+h-\frac{R^{2}}{d+h}){\left(\frac{R^{2}}{d+h}\right)}^{k-1}-(d-\frac{R^{2}}{d}){\left(\frac{R^{2}}{d}\right)}^{k-1}\}$$

$$+\frac{D\omega }{2}\frac{1+\kappa_{2}}{c_{3}}\frac{1}{k}[\frac{1}{d^{k}}-\frac{1}{{\left(d+h\right)}^{k}}],\;B_{-k}=\frac{D\omega }{2}\frac{1+\kappa_{2}}{\kappa_{2}c_{5}}\{\frac{1}{k}[{\left(\frac{R^{2}}{d+h}\right)}^{k}-{\left(\frac{R^{2}}{d}\right)}^{k}]-h\delta_{1k}+(d+h-\frac{R^{2}}{d+h}){\left(\frac{R^{2}}{d+h}\right)}^{k-1}-(d-\frac{R^{2}}{d}){\left(\frac{R^{2}}{d}\right)}^{k-1}\}$$

$$+\frac{D\omega }{2}\frac{c_{6}}{c_{5}}\frac{R^{2k}}{k}[\frac{1}{d^{k}}-\frac{1}{{\left(d+h\right)}^{k}}]$$

$$D=\frac{\mu_{2}}{2\pi (1-\nu_{2})},a=\frac{2\mu^{0}+\lambda^{0}-\tau^{0}}{4R\mu_{1}},b=\frac{2\mu_{1}(2\mu^{0}+\lambda^{0}-\tau^{0})}{4R\mu_{1}(\mu_{1}+\lambda_{1})},c_{1}=\frac{\mu_{1}(1+\kappa_{2})}{\mu_{2}\kappa_{1}+\mu_{1}(1+b)},\;c_{2}=\frac{\mu_{1}-\mu_{1}(1+b)}{\mu_{2}\kappa_{1}+\mu_{1}(1+b)}$$

$$c_{3}=1+(a+b)(k+1)+\frac{\mu_{2}\kappa_{1}}{\mu_{1}}+\frac{a(a+b)(1+k)(1-k)}{1+(k-1)a+\mu_{2}/(\kappa_{2}\mu_{1})},c_{4}=\frac{a(1+\kappa_{2})(1+k)}{\kappa_{2}[1+(k-1)a+\mu_{2}/(\kappa_{2}\mu_{1})]}$$

$$c_{5}=1+a(k-1)+\frac{\mu_{2}}{\mu_{1}\kappa_{2}}+\frac{a(a+b)(1+k)(1-k)}{1+(k+1)(a+b)+\mu_{2}\kappa_{1}/\mu_{1}}$$

$$c_{6}=\frac{\left(a+b)(1+\kappa_{2})(1-k\right)}{1+(k+1)(a+b)+\mu_{2}\kappa_{1}/\mu_{1}},c_{7}=1+a+\frac{\mu_{2}}{\mu_{1}\kappa_{2}}-\frac{3a(a+b)}{1+3(a+b)+\mu_{2}\kappa_{1}/\mu_{1}}$$

$$c_{8}=1+3(a+b)+\frac{\mu_{2}\kappa_{1}}{\mu_{1}}-\frac{3a(a+b)}{1+a+\mu_{2}/(\kappa_{2}\mu_{1})}$$

$c_{9}=\frac{3a(1+\kappa_{2})}{\kappa_{2}[1+a+\mu_{2}/(\kappa_{2}\mu_{1})]}$ and $\delta_{ij}$ is the Kronecker delta.

With Equations (8) and (9) substituted into formulas (7), we obtain the following necessary condition for nanocrack growth: $q>q_{c}$, where

(12) $$q=\frac{8\mu_{2}}{(2\gamma -\gamma_{b}){\left(D\omega \pi \right)}^{2}L}[{\left(\int_{0}^{L}\sigma_{yy}\sqrt{\frac{x}{L-x}}dx\right)}^{2}+{\left(\int_{0}^{L}\sigma_{xy}\sqrt{\frac{x}{L-x}}dx\right)}^{2}]$$

(13) $$q_{c}=\frac{32\pi (1-\nu )}{\omega^{2}}$$

## 4. Results and Discussion

Utilizing Equations (12) and (13), the influence of nanoinhomogeneity on nanocrack nucleation and propagation in the nanocrystalline matrix can be evaluated in detail. We use the following parameter value for the nanocrystalline Ni matrix: $\mu_{2}=79\mathrm{Gpa}$, $\upsilon_{2}=0.31$, $\gamma =1.725{J/m}^{2}$, $\gamma_{b}=0.69{J/m}^{2}$ [23]. We give α=π/4, because the value of α=π/4 corresponds to the direction of the maximum shear stress (induced by the external tensile load) at which the nanotwin generation is most favorable. In addition, we define the intrinsic lengths $\beta =\mu^{0}/\mu_{1}$, $\eta =\lambda^{0}/\mu_{1}$ and $\chi =\tau^{0}/\mu_{1}$. According to results in Miller and Shenoy [43], the absolute values of the intrinsic lengths β, η, and χ are nearly 0.1 nm.

### 4.1. Influence of Nanoinhomogeneity Shear Modulus on Nanocrack Nucleation

**Figure 2:**
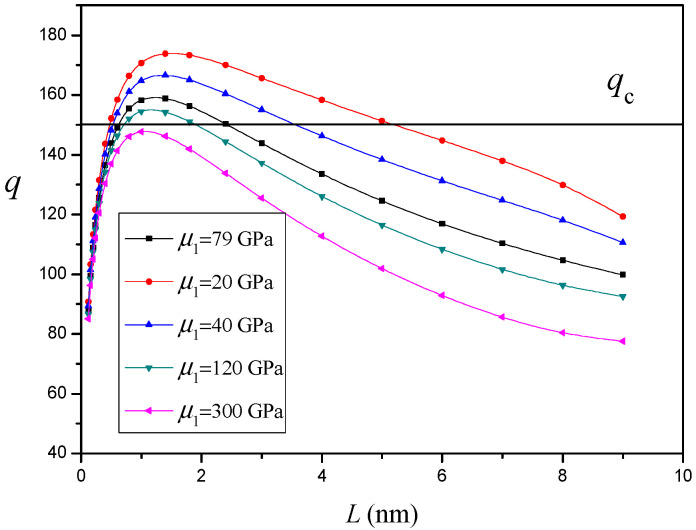
The variation of q versus nanocrack length L for different values of nanoinhomogeneity shear modulus $\mu_{1}$ (d=60nm and R=50nm).

The variation of q versus the nanocrack length L for different values of the nanoinhomogeneity shear modulus $\mu_{1}$ (d=60nm and R=50nm) without an interface effect is depicted in Figure 2. The horizontal line in Figure 2 shows the value of $q_{c}$. When the curve q lies higher than the horizontal line $q_{c}$, the nanocrack will grow. The critical nanocrack length $L_{c}$ and equilibrium nanocrack length $L_{e}$ correspond to the left and right intersection points of the curve q with the horizontal line $q_{c}$, respectively. A nanocrack is generated when the crack length reaches its critical value $L_{c}$. Then the nanocrack grows until its length reaches the equilibrium value $L_{e}$. It is shown in Figure 2 that, compared with the one-phase case ($\mu_{1}=\mu_{2}$), the stiff nanoinhomogeneity ($\mu_{1}>\mu_{2}$) increases the critical nanocrack length $L_{c}$ and decreases the equilibrium nanocrack length $L_{e}$, while the soft nanoinhomogeneity ($\mu_{1}<\mu_{2}$) would lead to the opposite situation. This indicates the stiff nanoinhomogeneity suppresses nanocrack nucleation and propagation, while soft nanoinhomogeneity promotes nanocrack nucleation and propagation. In particular, when the shear modulus of the nanoinhomogeneity is large enough ($\mu_{1}>212\mathrm{Gpa}$), the nanocrack is no longer generated.

### 4.2. Influence of Nanoinhomogeneity Radius and Position on Nanocrack Nucleation

**Figure 3:**
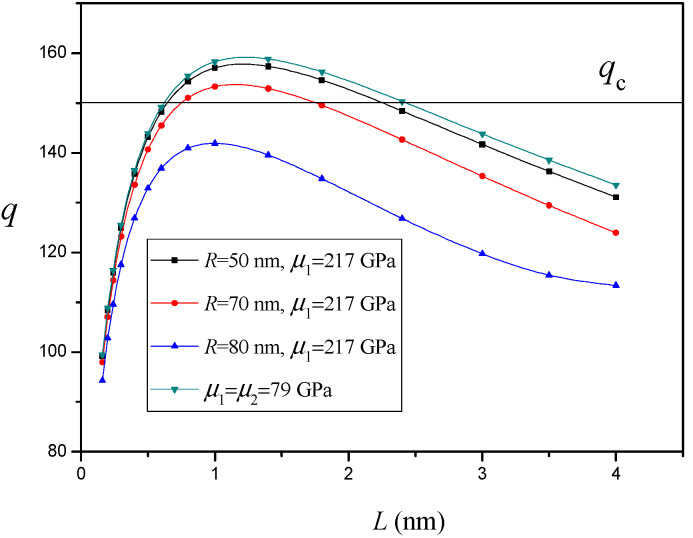
The variation of q versus nanocrack length L for different values of SiC nanoinhomogeneity radius R (h=2.7nm and d=85nm).

In this section, we focus on the case of Ni/SiC (metal-ceramic) nanocomposites. In the case of SiC nanoinhomogeneity, we obtained $\mu_{1}=217\mathrm{Gpa}$, $\upsilon_{1}=0.23$ [50]. The variation of q versus the nanocrack length L for different values of the SiC nanoinhomogeneity radius R (h=2.7nm and d=85nm) without an interface effect is depicted in Figure 3. It is shown that the SiC nanoinhomogeneity in the nanocrystalline Ni matrix increases the critical nanocrack length $L_{c}$ and decreases the equilibrium nanocrack length $L_{e}$. This indicates the SiC nanoinhomogeneity suppresses nanocrack nucleation and propagation [33]. As the SiC nanoinhomogeneity radius increases, the effect of the SiC nanoinhomogeneity will continue to grow stronger. In particular, when R>75nm, the nanocrack is no longer generated. The size of the second phase can be controlled in nanocomposites, so the analytical results could serve as a guide for the design of nanocomposites.

**Figure 4:**
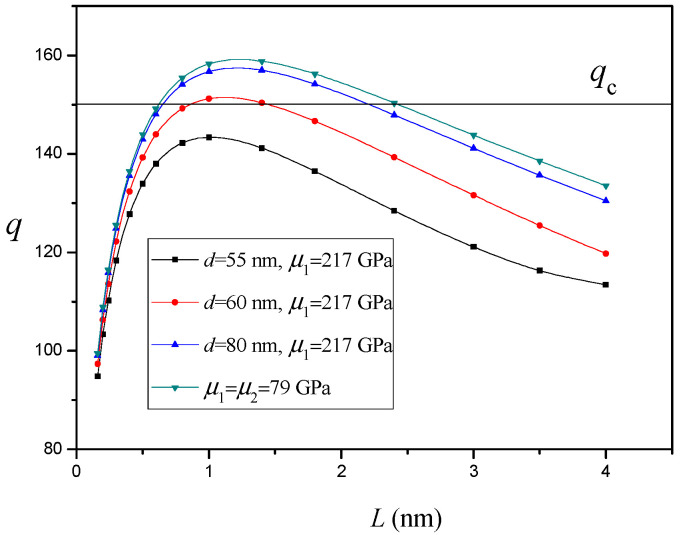
The variation of q versus nanocrack length L for different values of distance d between twin and SiC nanoinhomogeneity (h=2.7nm and R=50nm).

The variation of q versus the nanocrack length L for different values of distance d between the twin and SiC nanoinhomogeneity (h=2.7nm and R=50nm) without an interface effect is depicted in Figure 4. It is shown that, as the SiC nanoinhomogeneity approaches the twin, the effect of the SiC nanoinhomogeneity will continue to grow stronger. Especially, for a small enough value of d (d<58nm), the nanocrack is no longer generated. Figure 3 and Figure 4 clearly demonstrate that the SiC nanoinhomogeneity in the nanocrystalline Ni matrix suppresses nanocrack nucleation and propagation and thereby improves the tensile ductility of nanocomposites without loss of their predominantly high strength. The experimental results [30,33] demonstrate SiC nanoparticles can improve the ductility and tensile strength of the nanocrystalline Ni matrix. Lari Baghal et al. [33] consider the improvement to be ascribed to the suppressing effect of SiC nanoparticles on nanocrack propagation that can delay the fracture of the nanocomposites, which is consistent with our analytical result.

### 4.3. Influence of Interface Effect on Nanocrack Nucleation

**Figure 5:**
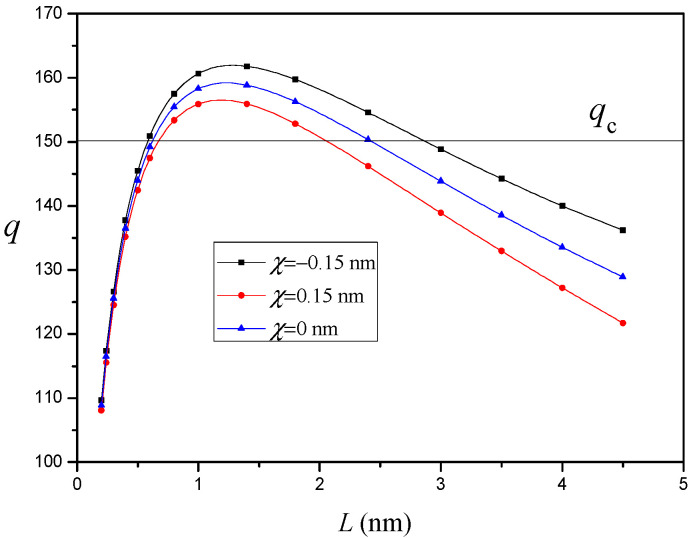
The variation of q versus nanocrack length L for different values of interface residual tension χ (d=15nm and R=10nm).

The variation of q versus the nanocrack length L for different values of the interface residual tension χ (d=15nm and R=10nm) is depicted in Figure 5. It is shown that if the interface residual tension is positive (χ>0), it will increase the critical nanocrack length $L_{c}$ and decrease the equilibrium nanocrack length $L_{e}$; if the interface residual tension is negative (χ<0), it will decrease the critical nanocrack length $L_{c}$ and increase the equilibrium nanocrack length $L_{e}$. This indicates that the positive interface residual tension suppresses nanocrack nucleation and propagation, while the negative interface residual tension promotes nanocrack nucleation and propagation. Furthermore, the effect of interface residual tension is rather significant.

**Figure 6:**
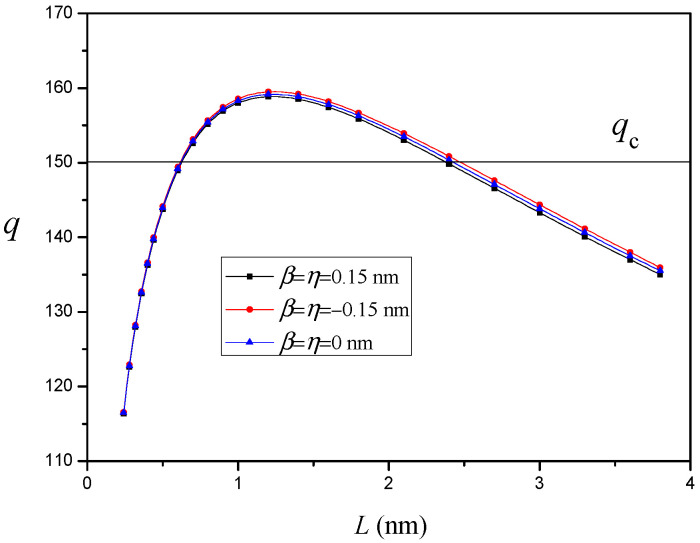
The variation of q versus nanocrack length L for different values of interfacial elastic constants β and η (d=15nm and R=10nm).

The variation of q versus the nanocrack length L for different values of interface elastic constants β and η (d=15nm and R=10nm) is depicted in Figure 6. It can be seen that the positive interface elastic constants increase the critical nanocrack length $L_{c}$ and decrease the equilibrium nanocrack length $L_{e}$, but the negative interface elastic constants would lead to the opposite result. This indicates that the positive interface elastic constants suppress nanocrack nucleation and propagation, which means the local hardening at the interface occurs due to the positive interface elastic constants. On the other hand, the negative interface elastic constants promote nanocrack nucleation, and local softening at the interface is produced. However, the interface elastic constants have a weak effect on nanocrack nucleation and propagation.

### 4.4. Influence of External Stress on Nanocrack Nucleation

**Figure 7:**
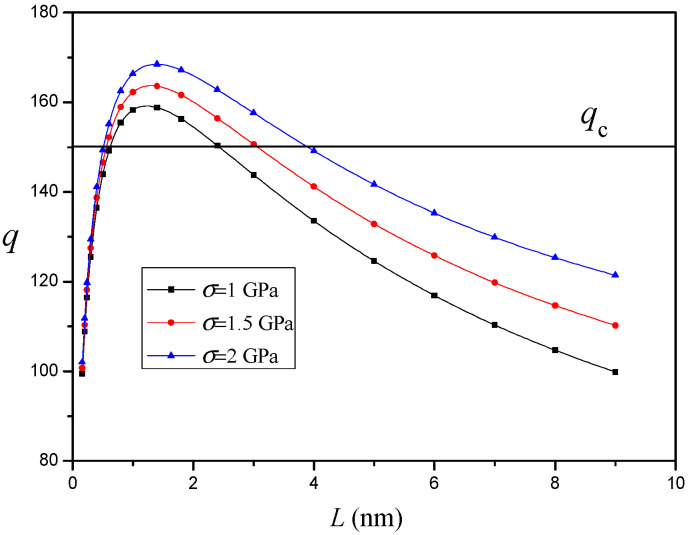
The variation of q versus nanocrack length L for different values of external stress σ ($\mu_{1}=\mu_{2}$).

The variation of q versus the nanocrack length L for different values of external stress σ ($\mu_{1}=\mu_{2}$) without an interface effect or nanoinhomogeneity is depicted in Figure 7. It is shown that, as the external stress σ increases, the critical nanocrack length $L_{c}$ decreases and the equilibrium nanocrack length $L_{e}$ increases. This result seems natural since the external stress σ drives nanocrack nucleation and propagation [23].

## 5. Concluding Remarks

In this paper, we have theoretically studied the nanoinhomogeneity effect on nanocrack nucleation and propagation at the deformation twin stopped by GBs in nanocomposite materials. The influence of the nanoinhomogeneity shear modulus, the nanoinhomogeneity radius, the nanoinhomogeneity position, the interface stress, and the external stress on nanocrack nucleation and propagation was investigated in detail. The results indicate that:

(1) The stiff nanoinhomogeneity suppresses nanocrack nucleation and propagation, while the soft nanoinhomogeneity promotes nanocrack nucleation and propagation. In particular, when the shear modulus of the nanoinhomogeneity is large enough, the nanocrack is no longer generated. Therefore, when manufacturing nanocomposites, we can choose the appropriate shear modulus of the particle to ensure that, as far as possible, the nanocrack is not generated, thereby improving the ductility of nanocomposites.

(2) The SiC nanoinhomogeneity in the nanocrystalline Ni matrix suppresses nanocrack nucleation and propagation and thereby improves the tensile ductility of nanocomposites without loss of their predominantly high strength. The analytical results are consistent with the corresponding experimental data [30,33].

(3) As the SiC nanoinhomogeneity radius increases and the SiC nanoinhomogeneity approaches the twin, the effect of the SiC nanoinhomogeneity on nanocrack nucleation and propagation will continue to grow stronger. In particular, when the nanoinhomogeneity radius is more than a certain value, the nanocrack is no longer generated. The size of the particle can be controlled in nanocomposites, so the analytical results could serve as a guide for the design of nanocomposites.

(4) Both the positive interface residual tension and the interface elastic constants suppress nanocrack nucleation and propagation, while the negative interface residual tension and the interface elastic constants promote nanocrack nucleation and propagation. Furthermore, the effect of the interface residual tension is rather significant. However, the interface elastic constants have a weak effect on nanocrack nucleation and propagation.

(5) When the external stress increases, the nanocrack generates and grows more easily.

## Data Availability

Not applicable.
